# lncRNA MSTRG.29039.1 Promotes Proliferation by Sponging hsa-miR-12119 via JAK2/STAT3 Pathway in Multiple Myeloma

**DOI:** 10.1155/2021/9969449

**Published:** 2021-08-11

**Authors:** Zhaoyun Liu, Mei Han, Nanhao Meng, Jingyi Luo, Rong Fu

**Affiliations:** Department of Hematology, Tianjin Medical University General Hospital, Tianjin 300052, China

## Abstract

Noncoding RNA (ncRNA) is involved in the occurrence, development, metastasis, and drug resistance of tumors and involves a variety of biological functions. In addition, miRNA can regulate proliferation and migration and even regulate epigenetics to promote the development of multiple myeloma (MM). However, the mechanism of ncRNA involved in MM is still unclear, and there are many unknown ncRNAs to be explored. This research is aimed at discovering the unknown lncRNA in MM through high-throughput sequencing and to study the mechanism and role of competitive endogenous RNA (ceRNA) involved in the pathogenesis of MM for the development of novel molecular markers and potential new targeted drugs. We screened out 262 new lncRNAs with statistical differences by RNA sequencing and selected the lncRNA MSTRG.29039.1 according to the expression and function of lncRNAs and their target genes in MM. We verified that MSTRG.29039.1 and its target gene OSMR were highly expressed in MM. After knockdown of MSTRG.29039.1 in MM cell lines, the expression of OSMR was decreased, and the expression of hsa-miR-12119 was upregulated which can also promote cell apoptosis and inhibit proliferation. Then, we knocked down hsa-miR-12119 and MSTRG.29039.1, we found that apoptosis of MM cells was reduced, and cell proliferation was increased compared with just knocking down hsa-miR-12119. We further verified the direct binding relationship between MSTRG.29039.1 and OSMR by the dual-luciferase reporter assay system. Thus, MSTRG.29039.1 can competitively bind with miRNA to counteract the inhibitory effect of miRNA on OSMR, which regulates cell proliferation and apoptosis through the JAK2/STAT3 pathway. In a conclusion, lncRNA MSTRG.29039.1 could promote proliferation by sponging hsa-miR-12119 via the JAK2/STAT3 pathway in multiple myeloma. This may be a molecular marker and a potential therapeutic target for MM.

## 1. Introduction

Multiple myeloma (MM) is a plasma cell disease characterized by biological heterogeneity whose clinical features are clonal hyperplasia of bone marrow plasma cells and accompanied by excessive production of monoclonal immunoglobulins, leading to a series of damages such as abnormal kidney function, hypercalcemia, anemia, and bone disease [1]. With the application of proteasome inhibitors, autologous stem cell transplantation (ASCT), immunomodulators, monoclonal antibodies, cellular immunity, and other immunotherapies, MM patients get deeper remission and prolong survival, while there are still some patients who cannot be healed, and even many patients still relapse [2]. Therefore, it is essential to study the potential pathogenesis of multiple myeloma.

With the rapid development of high-throughput technologies, we have gradually discovered that noncoding RNA (ncRNA) plays an important role in the pathogenesis of myeloma. Long noncoding RNA (lncRNA) is RNA that is more than 200 nucleotides in length and has little or no ability to synthesize proteins [3]. A large number of studies have shown that lncRNA is also involved in the occurrence, development, metastasis, and drug resistance of tumors. lncRNA involves a variety of biological functions and participates in the composition and regulation of cells, such as participating in cell differentiation, proliferation, DNA replication, RNA transcription, protein modification, nuclear transport, and other important functions [4–7]. MicroRNA (miRNA) is a short noncoding RNA that is approximately 22 nucleotides in length and regulates gene expression at the posttranscriptional level, usually inhibits the expression of target genes, and has been shown to be dysfunctional or abnormal in various cancers [8, 9]. A large number of experiments have shown that miRNA can regulate proliferation and migration and even regulate epigenetics to promote the development of MM [9, 10]. Recent experiments have proposed that ceRNAs are transcripts that can be mutually regulated at the posttranscriptional level by competitive binding to miRNAs [11]. CeRNAs can be all of the transcriptions with the miRNA binding sites (also called miRNA response elements (MRE)), including miRNA, lncRNA, rRNA, tRNA, pseudogene RNA, and circular RNA [12]. The mechanism of ceRNA is that two transcripts containing the same MRE are competitively binding to miRNA. When one transcript is upregulated, it will bind more miRNA, thereby inhibiting the binding of miRNA to the other transcript and reducing the inhibitory function [13]. Increasing experiments have proved that the content and activity of ceRNAs are dysregulated in tumors [14, 15], and artificial miRNA sponges containing multiple MREs have been used to inhibit oncogenic miRNAs and have shown tumor suppression functions [16, 17]. Therefore, in this experiment, we further studied the biological function of ncRNA in MM and its pathogenic mechanism through high-throughput sequencing, and we also predicted the interaction pathway of lncRNA-miRNA mRNA. These observations may provide support for the potential treatment and novel molecular markers of MM.

## 2. Materials and Methods

### 2.1. Patient and Cell Samples

Bone marrow was collected from 30 healthy volunteers as well as 37 newly diagnosed MM (NDMM) patients diagnosed in the General Hospital of Tianjin Medical University. There were 37 patients with NDMM in the group, including 22 males and 15 females, with a median age of 63 years (39-96 years). Among them, 22 patients with NDMM were less than 65 years old (59.45%), and 15 patients were over 65 years old (40.55%). The normal control group consisted of 30 healthy controls who matched the age and sex of the NDMM group, with a male-to-female ratio of 17/13 and a median age of 54 years (30-84 years) (Table 1). And then CD138 cells were sorted and purified. The specific operation was as follows: extract mononuclear cells from 5 ml bone marrow from patients and volunteers. And then the CD138 antibody and cells were thoroughly mixed and incubated at 4°C for 20 min in the dark. After rinsing the MS column fixed on the magnet with 500 *μ*L PBS twice, added the cell suspension to collect the negative cells. Wash 1-2 times with 500 *μ*L PBS buffer as well as removing the MS column from the magnetic field before adding 500 *μ*L PBS (Beijing solarbio science & technology, China) buffer and quickly beating it into the collection tube. At last, count the number of cells and add 700 *μ*L of TRIzol (Invitrogen, Carlsbad, CA, USA) per 10^7^ cells, storing the specimen at -80°C for later RNA extraction. We selected the CD138 cells of 2 healthy volunteers and 8 NDMM patients to operate high-throughput sequencing. All patients and volunteers included in the study signed an informed consent form and recorded their clinical characteristics. The study was approved by the local ethics committee. The collection of the above samples was reviewed and approved by the Ethics Committee of Tianjin Medical University General Hospital, with informed consent from both patients and healthy controls and signed informed consent voluntarily.

### 2.2. RNA and DNA Extraction and Real-Time Fluorescent Quantitative PCR

We separated RNA by TRIzol, chloroform, isoamyl alcohol, and ice ethanol (Tianjin Chemical Reagent Factory) and used fast reverse transcription kit (Tiangen Biotech, Beijing) to perform reverse transcription to obtain complementary DNA. Then, we prepared a reverse transcription reaction system (total volume was 20 *μ*L). The real-time fluorescent quantitative PCR system was 25 *μ*L, and the reaction was carried out under the following conditions: 95°C, 3 min; 95°C, 5 s, annealing temperature. Primer sequences were shown in Table 2 (Sangon Biotech, Shanghai). Perform three replicate measurements.

### 2.3. High-Throughput Sequencing and Identification of Differentially Expressed lncRNA

The experimental sequencing process was as follows: after extracting the total RNA of the sample, the rRNA was removed using a ribosomal kit, and the RNA was fragmented (the average fragment length is about 200 nt). Reverse transcription to synthesize single-stranded cDNA before synthesizing and purifying double-stranded cDNA and then repaired the ends and added adaptor primers to amplify and purify by PCR. Finally, we performed quality inspection on the library and sequence on the computer. For the detected mRNA, we further calculated the gene expression and analyzed the differentially expressed genes between samples and perform Gene Ontology (GO) analysis and KEGG biological pathway enrichment analysis on the differentially expressed genes. For the detected lncRNA, analyzed the differential expression of lncRNA between samples and gave specific annotations to perform the corresponding identification of ORF protein domain, coding potential, secondary structure prediction, and family analysis. The potential *cis*-regulated target gene of lncRNA was obtained by integrating the differential lncRNA with its neighboring (10 kb) differential mRNA data, while for the prediction of transregulation, firstly, extracted the sequence of differentially expressed lncRNA and mRNA, then used blast software for initial screening (*e* < 1*E* − 5), and at last used RNAplex software to screen again to identify possible target genes of lncRNA.

### 2.4. Prediction of miRNA and Its Target Genes

The three software miRanda, PITA, and RNAhybrid (Ruibo Biotechnology, China) were used to predict candidate miRNAs that interacted with lncRNA and their targeted regulated genes. The prediction of the analysis of interaction with lncRNA mainly considered the following 4 points: [1] the degree of perfect complementary pairing of the miRNA seed region, [2] MRE (miRNA recognition elements) sequence conservation, [3] the binding free energy of miRNA-lncRNA duplex, and [4] the sequence characteristics of the target molecule are determined. We predicted the recognition region of lncRNA and miRNA by miRanda, PITA, and RNAhybrid to identify the lncRNA that interacted with miRNA.

### 2.5. Cell Culture

Human MM cell lines (U266, OPM2, MM1S, ARD, KMS-11, and AMO-1 cells) (Peking Union Cell Bank, China) were cultured in RPMI-1640 medium (Solarbio, Beijing, China) and added with 10% fetal bovine serum (FBS, Gibco, California, USA) and 1% chain penicillin (Gibco), placed in a humid 37°C cell incubator with 5% CO_2_.

### 2.6. Cell Transfection

We inoculated the logarithmic growth of MM cells into a 6-well plate with approximately 1 − 2 × 10^5^ cells per well. Use Lipofectamine 3000 (Thermo Fisher Scientifi, China) reagent to perform cell transient transfection according to the manufacturer's instructions. For si-RNA, miRNA-inhibitor, fluorescence, and negative control were purchased from GenePharma (Table 2). Perform three replicate measurements.

### 2.7. Cell Proliferation

Use Cell Counting Kit-8 (Beyotime, China) for measurement. Inoculate 10^5^ MM cells/well into a 96-well plate and add 10 *μ*L CCK-8 reagent. After 4 hours of incubation in a cell incubator at 37°C and 5% CO_2_, the absorbance (*A*) at 450 nm wavelength was measured using the automatic microplate reader, and the cell proliferation ability was expressed as *A* value. Each group had 5 replicate wells, and each experiment was repeated 3 times. Cell survival rate = (experimental well OD value − blank well OD value)/(control well OD value − blank well OD value) × 100%.

### 2.8. Apoptosis

Washing the MM cells collected after transfection with 1 : 9 configured phosphate buffer solution (PBS; Corning Life Sciences, China), collect the cells after centrifugation at 1500 rpm for 5 minutes. Follow the instructions of the PE Annexin V cell apoptosis detection kit (BD Bioscience, USA). Finally, the apoptotic cells were distinguished by flow cytometry, and the apoptosis rate was calculated. Perform three replicate measurements.

### 2.9. Dual-Luciferase Reporter Assay

The MSTRG.29039.1 fragment with the predicted binding site of hsa-miR-12119 was synthesized and cloned into the luciferase reporter gene to form the reporter vector MSTRG.29039.1-wild-type (MSTRG-WT). MSTRG.29039.1-hsa-miR-12119 binding site was mutated as instructed and named MSTRG.29039.1 mutant (MSTRG-MUT). OSMR-wild-type (OSMR-WT) and OSMR-mutated type (OSMR-MUT) were also obtained in accordance with the above method. MSTRG-WT/OSMR-WT/MSTRG-MUT/OSMR-MUT/miRNA-mimics/miRNA-NC (Hunan Fenghui Biotech, China) were transfected into HEK-293T cells according to the above transfection method. 48 hours after transfection, the dual life luciferase assay system (Nanjing Nuowizan Biotech, China) was applied according to the prescribed procedure. Perform three replicate measurements.

### 2.10. Western Blot

After 72 h of transfection, the cells were collected into 1.5MLEP tubes. The cells were lysed with protein lysates consisting of RIPA: PMSF = 100 : 1. The protein concentration was determined using the BCA protein assay kit. After adding the protein sample buffer, the cells were boiled in a 100°C- water bath for 10 min to denaturate the proteins.

Start electrophoresis after adding protein and maker to the sample tank. After electrophoresis, the proteins were transferred to a polyvinylidene difluoride (PVDF) membrane (Solarbio, Beijing, China). PVDF membrane was cut according to the molecular weight of the required proteins. And then the PVDF membrane was sealed in 5% skimmed milk powder for 1 h and washed with TBST for 3 times. The antibody (GAPDH, OSMR, JAK2, STAT3, pJAK2, pSTAT3) (Cell Signaling Technology, USA) was incubated and placed in a shaker for 4°C overnight, and the anti-rabbit IgG sheep antibody (Cell Signaling Technology, USA) incubated for exposure the next day. The chromogenic condition of the protein was observed and photographed.

### 2.11. Statistical Analysis

Use SPSS software 22.0 and GraphPad Prism5.0 to complete the data analysis. All data were expressed as the mean ± SD obtained in three repeated determinations. The Mann-Whiney *U* test was used to compare the differences between the two groups. The Kruskal-Wallis *H* test was used for comparison between multiple groups. *P* < .05 was considered statistically significant.

## 3. Results

### 3.1. The Expression of Differential lncRNAs in MM

**Figure 1:**
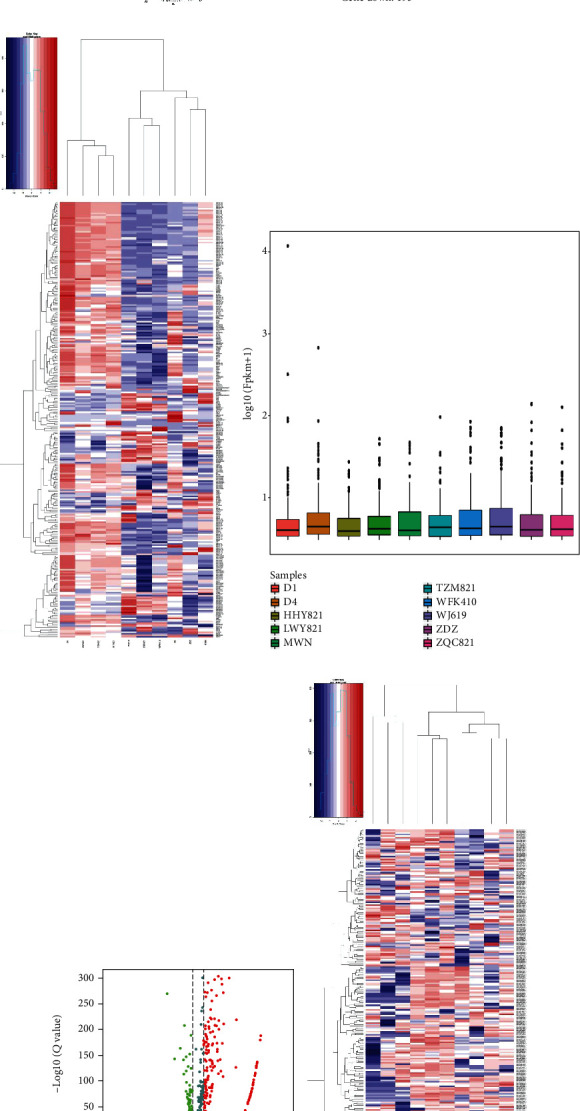
The expression of differential lncRNAs in MM: (a) The position of reads on the chromosome. The outer circle represents the position of the chromosome, the red inner circle represents the positive strand, and the green represents the negative strand. (b) Volcano map of differential gene expression analysis between samples. The abscissa represents the fold change of the gene expression in different samples; the ordinate represents the statistical significance of the difference in gene expression changes, red dots indicate significant upregulated genes, and green indicates significant downregulated genes. (c) Differential gene expression analysis between sample heat map. Red and green, respectively, represent upregulated and downregulated genes. (d) New lncRNA expression (FPKM). (e) Volcano map of the differential expression of new lncRNA. Red and green, respectively, represent upregulated and downregulated genes. (f) Heat map of the differential expression of new lncRNA. Red and green represent upregulated and downregulated genes, respectively.

High-throughput sequencing results showed a total of 44591 base sequences (Figure 1(a)). We based on *P* < 0.05 and fold change > 2 to elect the differentially expressed genes and detected 285 differential genes that were statistically different, 92 were upregulated, and 193 downregulated (Figures 1(b) and 1(c)); we used the same method to screen out 262 unknown lncRNAs with statistical differences, of which 203 were upregulated, and 59 were downregulated (Figures 1(d)–1(f)).

### 3.2. Screen Prediction of Differential lncRNA and Its Target Genes

**Figure 2:**
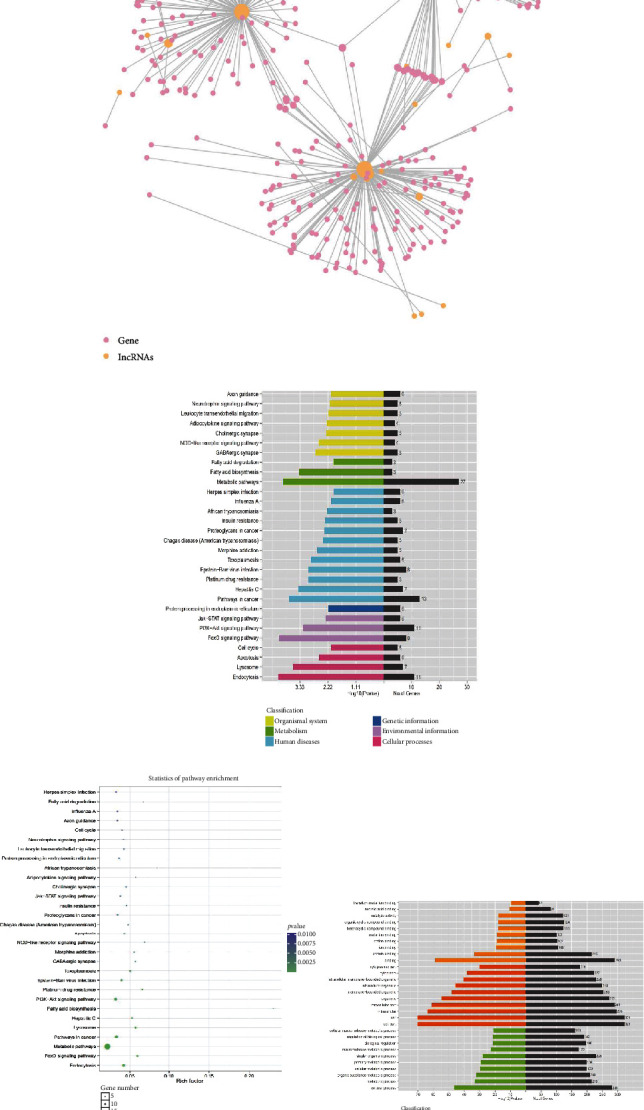
Screen prediction of differential lncRNA and its target genes. (a) Interaction analysis diagram of lncRNA and mRNA. The red dots represent lncRNA, the green dots represent their predicted mRNA, and the line represents the relationship between lncRNA and mRNA. (b) KEGG pathway diagram of the target gene. (c) KEGG pathway bubble diagram of the target gene. The abscissa indicates the ratio of enriched differential genes to the background genes of the pathway, and the ordinate indicates the name of the pathway; the size of the dot in the figure indicates the number of enriched differential genes, and the color indicates the *P* value. (d) GO pathway diagram of the target gene describes cell composition, molecular functions, and biological processes.

From the upregulated 203 lncRNAs, we selected 63 lncRNAs that were highly expressed in 8 myeloma patients and submitted them for testing to predict their target genes and the GO function of the target genes and KEGG biological pathway enrichment analysis (Figures 2(a)–2(d), Supplementary Table 1). Biological pathway analysis is based on the Kyoto Encyclopedia of Genes and Genomes (KEGG) biological pathway database. We calculated the *P* value by Fisher exact test (*P* < 0.05 is the significance threshold) to obtain signal transduction and disease pathways. This analysis was mainly concentrated in metabolism, human diseases, environmental information processing, and cellular processes, such as fatty acid biosynthesis, hepatitis C, various cancer pathways, FoxO signaling pathway, endocytosis,and lysosomes. Gene Ontology (GO) analysis can annotate the function of each gene and calculate the most significant function in a specific series of genes through statistical analysis such as hypergeometric distribution. This analysis mainly showed significant differences in cellular components, among which the cell part and intracellular part were the most significant.

### 3.3. The Unknown lncRNA MSTRG.29039.1 and Its Target Gene OSMR Were Highly Expressed in MM

**Figure 3:**
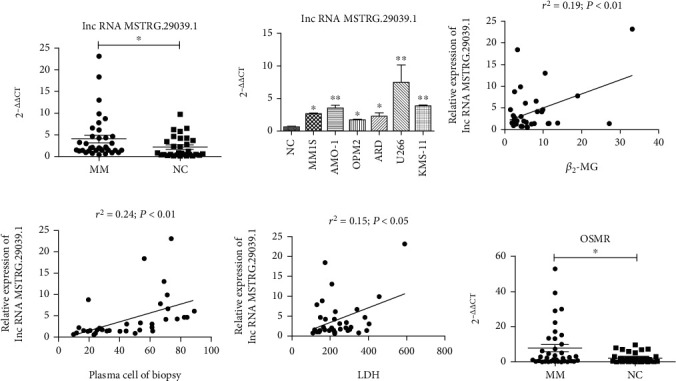
The unknown lncRNA MSTRG.29039.1 and its target gene OSMR were highly expressed in MM: (a) Detection of the relative expression of MSTRG.29039.1 in NDMM patients and healthy controls by real-time PCR. (b) The expression level of MSTRG.29039.1 in MM cell lines and controls. (c) Correlation analysis between MSTRG.29039.1 and *β*2-MG (*P* < .05). (d) Correlation analysis between MSTRG.29039.1 and plasma cell of biopsy. (e) Correlation analysis between MSTRG.29039.1 and LDH. (f) The relative expression of OSMR in NDMM patients and healthy controls (^∗^*P* < .05).

We selected lncRNA-MSTRG.29039.1 based on the target gene function and the expression level of unknown lncRNA in MM. Its expression level in patients with multiple myeloma was significantly higher than that in healthy controls. We compared the relative expression of lncRNA MSTRG.29039.1 in 37 NDMM and 30 healthy controls. The relative expression of MSTRG.29039.1 in MM patients was 4.082 ± 0.8207, while that of healthy controls was 2.158 ± 0.4519. The expression level in MM patients was higher than that in healthy controls (*P* < .05), and the expression level in MM cell lines (U266, AMO-1, KMS-11, MM1S, ARD, OPM2) was also significantly higher than that in healthy controls (Figures 3(a) and 3(b)). To explore the correlation between MSTRG.29039.1 and clinical, we collected the clinical data of patients and did a correlation analysis and found that the relative expression of MSTRG.29039.1 was not related to gender, age, and R-ISS staging (*P* > .05), while statistically significant with ISS staging (*P* < .05) (Table 1) and positive with *β*2-microglobulin (*β*2-MG) (*P* < .01), plasma cell of biopsy (*P* < .01), and lactate dehydrogenase (LDH)(*P* < .05) (Figures 3(c)–3(e)). Based on the results of target gene prediction, we found that its target gene OSMR was significantly highly expressed in MM, and it has been reported in the literature that OSMR can promote tumor cell proliferation, inhibit apoptosis, promote tumor metastasis, and promote tumor progression [18]. The relative expression of OSMR in MM patients and healthy controls detected by PCR was 7.872 ± 2.029 and 2.219 ± 0.5443, respectively. We found that compared with healthy controls, the expression of OSMR in MM was significantly higher (*P* < .05) (Figure 3(f)).

### 3.4. Knockdown MSTRG.29039.1 to Promote MM Cell Apoptosis and Inhibit Proliferation

**Figure 4:**
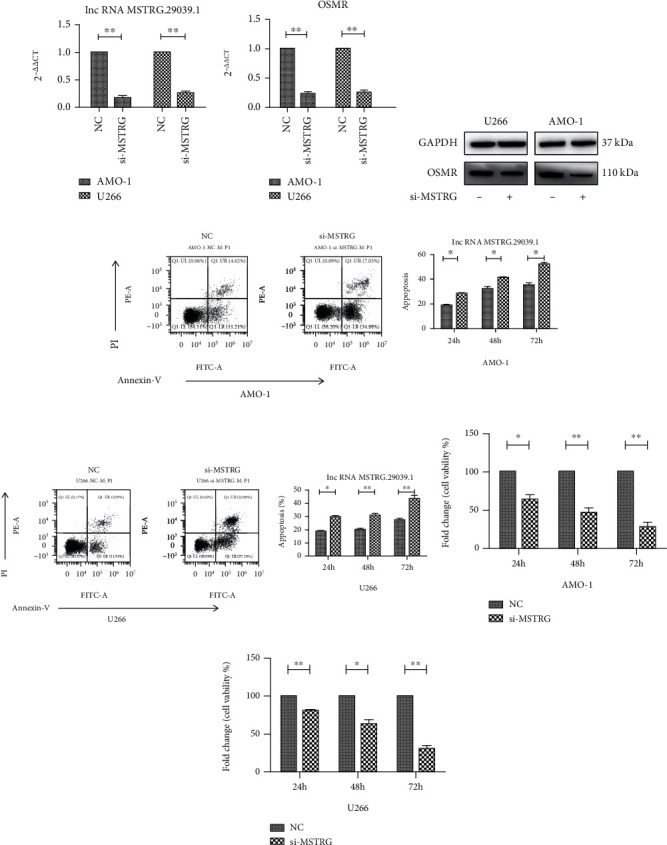
Knock down MSTRG.29039.1 to promote MM cell apoptosis and inhibit proliferation. (a) Transfection efficiency after knocking down MSTRG.29039.1 in AMO-1 and U266 cell lines. (b) The relative expression of OSMR after knocking down MSTRG.29039.1 in AMO-1 and U266 cell lines by PCR. (c) The expression of OSMR after knocking down MSTRG.29039.1 in AMO-1 and U266 cell lines by Western blot. (d) Flow cytometry and statistical graphs of apoptosis after knocking down MSTRG.29039.1 in AMO-1 cell line. (e) Flow cytometry and statistical graphs of apoptosis after knocking down MSTRG.29039.1 in U266 cell line. (f) Cell survival rate after knocking down MSTRG.29039.1 in AMO-1 cell line. (g) Cell survival rate after knocking down MSTRG.29039.1 in U266 cell line (^∗^*P* < 0.05, ^∗∗^*P* < 0.01).

To explore the role of MSTRG.29039.1 in MM, we used si-MSTRG to knock down MSTRG.29039.1 in U266 and AMO-1 cell lines. The transfection efficiency was first confirmed by real-time PCR (Figure 4(a)). PCR and Western blot, respectively, verified that the target gene OSMR was downregulated at both the mRNA level and the protein level, which may be positively regulated by MSTRG.29039.1 (Figures 4(b) and 4(c)). Subsequently, cell apoptosis was detected by flow cytometry. We found that apoptosis increased significantly after knocking down MSTRG.29039.1 in U266 cell line, and the apoptosis rate increased from 18.85 ± 0.76 to 29.79 ± 0.85 after 24 h of knockdown; at 48 h and 72 h, the apoptosis rates before and after knockdown were 20.08 ± 1.18 vs. 31.01 ± 1.33; 27.38 ± 1.36 vs. 43.60 ± 2.35). Similarly, similar results were observed in the AMO-1 cell line. The apoptosis rate raised after knocking down MSTRG.29039.1, and it was found to be time-dependent (NC vs si-MSTRG, 24 h: 19.06 ± 0.33 vs. 28.24 ± 1.00; 48 h: 32.51 ± 1.23 vs. 41.27 ± 0.75; 72 h: 35.29 ± 1.39 vs. 52.36 ± 1.09) (Figures 4(d) and 4(e)). We also tested the cell proliferation in the two cell lines after knocking down MSTRG.29039.1 and found that it was consistent with the above phenomenon. The low expression of MSTRG.29039.1 in the U266 and AMO-1 cell lines was related to the decrease of cell proliferation. In the U266 cell line, after 24 h of knockdown, the cell survival rate of the NC and si-MSTRG groups was 100% vs. 80.82% ± 1.42, the 48 h and 72 h cell survival rates were 100% vs. 63.04% ± 5.79 and 100% vs. 31.00% ± 3.37, and the cell survival rate in AMO-1 also gradually decreased with time (NC vs. si-MSTRG 24 h: 100% vs. 63.50% ± 6.50; 48 h: 100% vs. 46.23% ± 5.40; 72 h: 100% vs. 27.70% ± 5.79) (Figures 4(f) and 4(g)).

### 3.5. Knockdown OSMR Improves MM Cell Apoptosis and Reduces Proliferation

**Figure 5:**
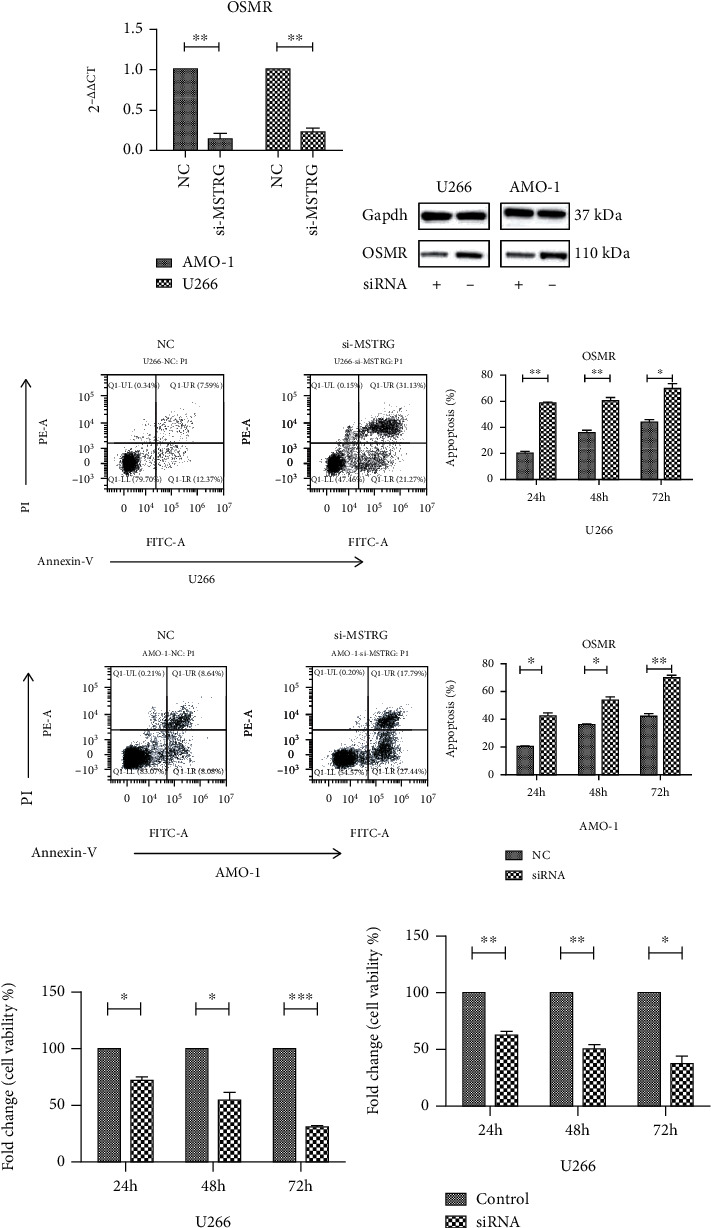
Knockdown OSMR improves MM cell apoptosis and reduces proliferation. (a) Transfection efficiency after knocking down OSMR in AMO-1 and U266 cell lines. (b) The expression of OSMR after knocking down OSMR in AMO-1 and U266 cell lines by Western blot. (c) Flow cytometry and statistical graphs of apoptosis after knocking down OSMR in U266 cell line. (d) Flow cytometry and statistical graphs of apoptosis after knocking down OSMR in AMO-1 cell line. (e) Cell survival rate after knocking down OSMR in U266 cell line. (f) Cell survival rate after knocking down OSNR in AMO-1 cell line (^∗^*P* < 0.05, ^∗∗^*P* < 0.01, ^∗∗∗^*P* < 0.001).

We found that in a variety of tumors such as pancreatic ductal adenocarcinoma, glioblastoma, and colorectal cancer, OSMR is highly expressed, and OSMR can regulate cell proliferation and tumor growth [19–21]. To study the role of OSMR in MM, in view of the increased expression of OSMR in MM, we decided to use siRNA to knock down OSMR in the U266 and AMO-1 cell lines. The transfection efficiency was confirmed by PCR (Figure 5(a)) and verified that the expression at the protein level was also decreased by Western blot (Figure 5(b)). Afterwards, we detected cell apoptosis by flow cytometry. We found that apoptosis increased significantly after knocking down OSMR in the U266 cell line. After knocking down for 24 hours, the apoptosis rate of untreated cells was 21.73% ± 2.05, while the apoptosis rate after using si-RNA was increased to 57.13% ± 1.04; the apoptosis rate of NC vs. si-RNA at 48 h and 72 h was 36.80% ± 1.36 vs. 66.20% ± 2.18; 38.32% ± 3.20 vs. 71.73% ± 3.06 (picture 5C). Similarly, we also observed similar results in the AMO-1 cell line. The apoptosis rate of the NC group was 21.69% ± 0.92 at 24 h, and the apoptosis rate of the si-RNA group increased to 41.57% ± 3.17; at 48 h, the apoptosis rate of the NC vs. si-RNA group was 36.26% ± 1.34 vs. 49.48% ± 1.74; 72 h: 38.31% ± 2.07 vs. 68.01 ± 1.54. After knocking down OSMR, the apoptosis rate of the two cell lines went up significantly, and it was time-dependent (Figure 5(d)). We also tested the cell proliferation in the two cell lines after knocking down OSMR and found that it was consistent with the increase in apoptosis rate. In the U266 and AMO-1 cell lines, the low expression of OSMR was related to the diminishment in cell proliferation. After 24 h of treatment, the cell survival rate of the NC and si-RNA groups in the U266 cell line was 100% vs. 71.67% ± 3.34, and the 48 h and 72 h cell survival rates were 100% vs. 53.63% ± 7.47, 100% vs. 30.46% ± 1.24 (Figure 5(e)). And the cell survival rate in AMO-1 also gradually drops with time (NC vs. si-RNA 24 h: 100% vs. 61.74% ± 3.83; 48 h: 100% vs. 50.26% ± 3.50; 72 h: 100% vs. 43.25% ± 6.38) (Figure 5(f)).

### 3.6. The Screening of miRNA and the Construction of lncRNA-miRNA-mRNA Interaction

**Figure 6:**
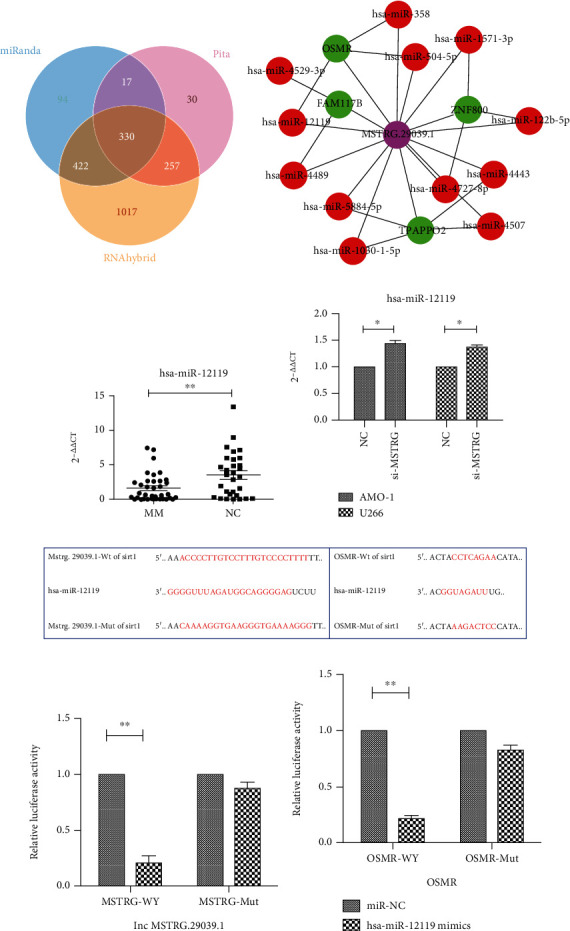
The screening of miRNA and the construction of lncRNA-miRNA-mRNA interaction. (a) Veen diagram of candidate miRNAs predicted to interact with lncRNA MSTRG.29039.1. (b) lncRNA-miRNA-mRNA interaction diagram. Pink circle is the new lncRNA, the red circle is miRNA, and the green circle is mRNA. (c). The relative expression of hsa-miR-12119 in NDMM patients and healthy controls by PCR. (d) The relative expression level of hsa-miR-12119 before and after knocking down MSTRG.29039.1. (e) The binding site of the wild type and mutant type of MSTRG.29039.1 and OSMR with hsa-miR-12119. (f) The dual luciferase assay report of MSTRG.29039.1 and hsa-miR-12119. (g) The dual luciferase assay report of OSMR and hsa-miR-12119.

As increasing lncRNAs are discovered, the complexity and diversity of potential ceRNA interactions have increased exponentially. Studies have found that some lncRNAs may be naturally effective spongy miRNAs in some cases [22]. We use miRanda, PITA, and RNAhybrid software to predict miRNAs interacting with MSTRG.29039.1 in four common authoritative databases: TargetScan, miRanda, CLIP-seq, and miRDB,and draw the Venn diagram, which shows a total of 330 target genes in the intersectional part (Figure 6(a)). We used the same method to predict the target gene of miRNA and screened out the same predicted target gene as MSTRG.29039.1 and drew the lncRNA-miRNA-mRNA interaction diagram (Figure 6(b)). From this, we can get that there are three miRNAs that meet the standard and can interact with MSTRG.29039.1 and OSMR. These three miRNAs are hsa-miR-12119, hsa-miR-504-5p, and hsa-miR-658 in turn. We further screened the expression of these three miRNAs in MM patients and healthy controls and the expression of miRNA after knockdown of MSTRG.29039.1 (Supplementary Figure 1). Finally, we found only hsa-miR-12119 lowly expressed in NDMM patients (NDMM vs. NC:1.612 ± 0.33 vs. 3.526 ± 0.5948) (*P* < .01, Figure 6(c)). And after knocking down MSTRG.29039.1 in AMO-1 and U266 cell lines, the expression of hsa-miR-12119 was significantly increased (AMO-1: *P* < .05; U266: *P* < .05, Figure 6(d)). This is in line with the assumption that lncRNA MSTRG.29039.1 maybe bind to hsa-miR-12119. Thereafter, we performed a dual luciferase experiment.  Figure 6(e) shows the binding sites of MSTRG-WT, MSTRG-MUT, OEMR-WT, and OSMR-MUT with hsa-miR-12119. The dual-luciferase reporter assay system shows that MSTRG.29039.1 (Figure 6(f)) and OSMR (Figure 6(g)) can be directly combined with hsa-miR-12119, which reveals that MSTRG.29039.1 can interact with OSMR as ceRNA and participate in the regulation of MM cell proliferation and apoptosis.

### 3.7. Knockdown of Hsa-miR-12119 Promotes MM Cell Proliferation

**Figure 7:**
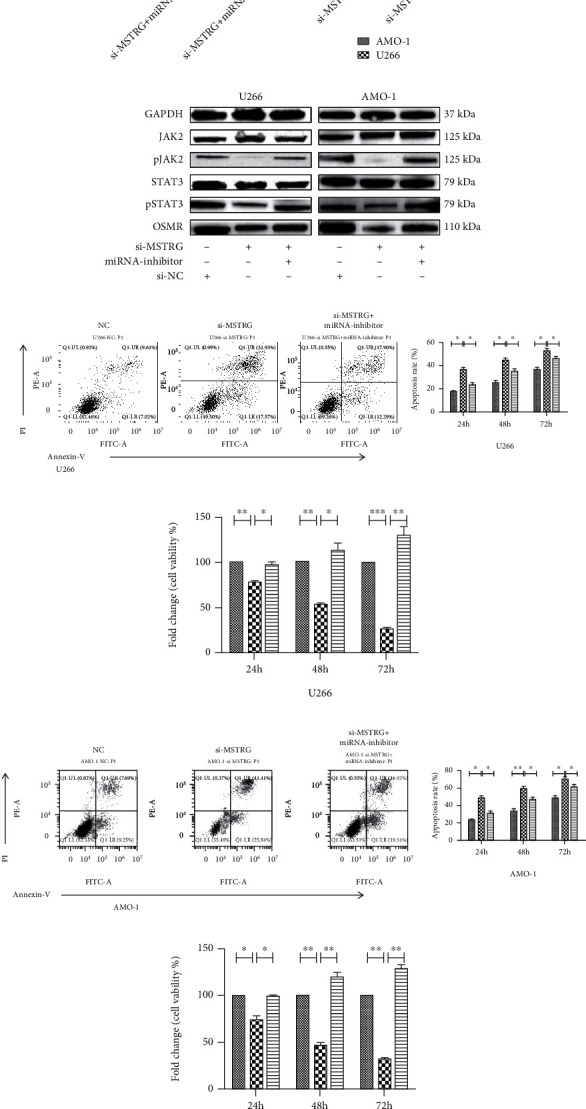
Knockdown of hsa-miR-12119 promotes MM cell proliferation. (a) The expression of OSMR after knocking down MSTRG and MSTRG+hsa-miR-12119 in AMO-1 and U266 cell lines by PCR. (b)The expression of hsa-miR-12119 after knocking down MSTRG and MSTRG+hsa-miR-12119 in AMO-1 and U266 cell lines by PCR. (c) The expression of OSMR/JAK2/STAT3/pJAK2/pSTAT3/GAPDH after knocking down MSTRG and MSTRG+hsa-miR-12119 in AMO-1 and U266 cell lines by Western blot. (d). Flow cytometric and statistical graphs of apoptosis after knocking down MSTRG and MSTRG+hsa-miR-12119 in U266 cell line. (e) Cell survival rate after knocking down MSTRG and si-MSTRG+ miRNA-inhibitor in U266 cell line. (f) Flow cytometric and statistical graphs of apoptosis after knocking down MSTRG and MSTRG+hsa-miR-12119 in AMO-1 cell line. (g) Cell survival rate after knocking down MSTRG and si-MSTRG+ miRNA-inhibitor in AMO-1 cell line (^∗^*P* < 0.05, ^∗∗^*P* < 0.01, ^∗∗∗^*P* < 0.001).

To further verify whether MSTRG.29039.1 sponges hsa-miR-12119 to counteract the inhibitory effect on OSMR, we further carried out a rescue experiment. Firstly, after knocking down MSTRG.29039.1 in the U266 and AMO-1 cell lines, we detected that the expression of hsa-miR-12119 increased at the RNA level, while the expression of OSMR decreased. After knocking down hsa-miR-12119 at the same time, the expression of hsa-miR-12119 (Figure 7(a)) decreased as expected, while the expression level of OSMR (Figure 7(b)) escalated; Western blot results also proved that the OSMR protein decreased after knocking down MSTRG.29039.1, and after knocking down hsa-miR-12119 at the same time, the expression of OSMR increased (Figure 7(c)).

We further studied whether knocking down hsa-miR-12119 can rescue the apoptosis and proliferation pathways regulated by OSMR. After knocking down MSTRG.29039.1, the apoptosis rate of the si-MSTRG group as described above was higher than that of the NC group, and the cell survival rate was reduced in the U266 and AMO-1 cell lines. In the U266 cell line, when we knocked down hsa-miR-12119 and MSTRG.29039.1 at the same time, we found that the apoptosis rate dropped from 34.51% ± 2.95 to 22.96% ± 2.01 at 24 h; at 48 h and 72 h, the apoptosis rate of the si-MSTRG group vs. si-MSTRG+miRNA-inhibitor group was 44.14% ± 3.02 vs. 32.76% ± 1.00 and 53.56% ± 2.32 vs. 39.69% ± 1.50 (Figure 7(d)). Compared with only knocking down MSTRG.29039.1, the cell survival rate at 24 h increased from 77.47% ± 1.81 to 97.33% ± 2.88 when knocking down MSTRG.29039.1 and hsa-miR-12119 at the same time; at 48 h and 72 h, the cell survival rate of the si-MSTRG vs si-MSTRG±miRNA-inhibitor group was 53.37% ± 2.19 vs. 112.64% ± 8.33 and 26.99% ± 1.94 vs. 129.44% ± 9.68 (Figure 7(e)). Similar results were obtained in the AMO-1 cell line. The apoptosis rate of knocking down MSTRG.29039.1 and hsa-miR-12119 at 24 h, 48 h, and 72 h was lower than that of just knocking down MSTRG.29039.1 and improve the cell survival rate (Figures 7(f) and 7(g)). Therefore, we believe that MSTRG.29039.1 can sponge hsa-miR-12119 and counteract the inhibitory effect on OSMR.

### 3.8. OSMR Regulates MM Cell Apoptosis and Proliferation via JAK2/STAT3 Pathway

Through the above experiments, we have gradually proved that MSTRG.29039.1 competes with OSMR to bind hsa-miR-12119, thereby reducing the inhibitory effect of hsa-miR-12119 on OSMR. OSMR can regulate cell apoptosis and proliferation, thereby affecting tumor cells and promoting tumor development. However, we still do not know how OSMR affects cell proliferation and apoptosis in MM.

The activation of JAK kinase can lead to phosphorylation and dimerization of tyrosine or serine residues in the c-terminal domain of the STAT3 protein. The activated STAT3 dimer enters the nucleus and initiates the transcription of target genes [23, 24]. And it has been found that JAK2/STAT3 regulates cell apoptosis and proliferation in MM [25]. We then think about whether OSMR may also regulate cell proliferation and apoptosis through the JAK2/STAT3 pathway. Therefore, we discuss the expression of key proteins in the JAK2/STAT3 pathway. We used Western blot to verify that after knocking down MSTRG.29039.1, and compared with the normal group, the protein levels of pJAK2 and pSTAT3 decreased, and the total protein levels of JAK2 and STAT3 did not change significantly. After knocking down MSTRG.29039.1 and hsa-miR-12119 at the same time, compared with knocking down MSTRG.29039.1, the protein levels of pJAK2 and pSTAT3 were significantly increased, and the total protein levels of JAK2 and STAT3 remained unchanged (Figure 7(c)). It proves that OSMR can regulate cell apoptosis and proliferation via the JAK2/STAT3 pathway and participate in the occurrence and development of tumors.

## 4. Discussion

Multiple myeloma is the second most common malignant tumor in the blood disease system. The standardized prevalence and incidence rates in our country are 5.68 (5.64-5.72) and 1.15 (1.11-1.19), respectively, while those in Western Europe and North America are even higher [26]. With the development of new treatments, the survival rate of myeloma patients has been significantly improved [27, 28], but drug resistance and relapse are still common in patients. Therefore, we need to further explore the pathogenesis of MM to discover new molecular markers and potential therapeutic targets. ncRNA has been found to participate in important biological processes such as chromatin remodeling, transcription, and posttranscriptional modification in various cancers by promoting and suppressing cancer [29]. lncRNA also plays an important role in various cancers, participating in part of the process of tumor proliferation, invasion, and metastasis [30, 31]. Our study found 63 new lncRNAs highly expressed in MM patients and predicted the interacting miRNAs and their target genes. To further explore the biological functions and metabolic pathways of lncRNA target genes, we performed the gene function (GO and KEGG analysis). These data reveal the underlying mechanism of the occurrence and development of MM and provide data support for us to find novel molecular markers and therapeutic targets. We verified that the new lncRNA MSTRG.29039.1 screened out is highly expressed in MM patients.

OSM is a member of the interleukin 6 (IL-6) receptor family, which is produced by monocytes/macrophages, dendritic cells, and T lymphocytes [32]. Oncostatin M receptor (OSMR) can bind to gp130 to mediate the biological functions of OSM [33, 34], and the combination of these two signals could play roles through ERK1/2, p38, JNK, PI3K/AKT, PKC*δ*, and the main JAK/STAT pathways [32]. In gastric cancer, OSM–OSMR can promote the proliferation, migration, invasion, and metastasis of tumor cells through the activation of STAT3/FAK/Src signals [18]. The overexpression of OSM and OSMR in cervical squamous cell carcinoma (SCC) can also cause a variety of malignant effects, including infiltration and production of angiogenic factors and associated with poor clinical outcomes [35]. We verified OSMR is highly expressed in MM. After knocking down MSTRG.29039.1, the expression of OSMR was decreased to promote MM cell apoptosis and inhibit proliferation. Therefore, MSTRG.29039.1 can be considered to play a carcinogenic role in the development of MM and can be regarded as a molecular indicator.

The abnormal expression of miRNA is also closely related to the disease [36, 37]. Of the three predicted miRNAs, MiR-658 is overexpressed in gastric cancer and induces gastric cancer metastasis by activating the PAX3-MET pathway [38], while miR-504 can inhibit cell proliferation and migration in non-small-cell lung cancer [39], oral squamous cell carcinoma [40], liver cancer [41], and overexpression in osteosarcoma [42], and breast cancer [43] to enhance the growth and metastasis of tumors. Therefore, it discloses miRNA that plays different roles in different tumors. However, there is little literature on hsa-miR-12119. We selected hsa-miR-12119 for subsequent studies by predicting the lncRNA-miRNA-mRNA interaction network and the expression of miRNA. Then, the rescue experiment was carried out to verify our hypothesis that MSTRG.29039.1 can sponge hsa-miR-12119 and counteract the inhibition of the OSMR protein. In the two MM cell lines of AMO-1 and U266, the apoptosis rate of knocking down MSTRG.29039.1 and hsa-miR-12119 at the same time is lower than that of just knocking down MSTRG.29039.1, while the cell survival rate is increasing. It is believed that MSTRG.29039.1 may interact with OSMR as a ceRNA and participate in the regulation of MM cell proliferation and apoptosis. Next, we verified by Western blot that OSMR can regulate cell apoptosis and proliferation via JAK2/STAT3 and participate in the occurrence and development of MM.

In summary, our results indicate that MSTRG.29039.1 is highly expressed in MM patients and can potentially competitively bind to hsa-miR-12119, leading to the overexpression of OSMR, reducing apoptosis, and promoting proliferation. We discovered the novel lncRNA MSTRG.29039.1 and found that it plays an important role in the occurrence and development of MM, and it is expected to become a molecular marker and potential therapeutic target for MM.

## Figures and Tables

**Table 1:** Baseline characteristics of MM patients.

	NDMM *n* (*n*/*N*%)	Relative expression of lncRNA MSTRG.29039.1	*P* value
*N*	37	3.553 ± 0.553	
Gender			
Male	22 (59.45)	4.08 ± 1.28	0.8649
Female	15 (40.55)	2.92 ± 0.75	
Age			
<65	22 (59.45)	4.22 ± 1.14	0.2859
≥65	15 (40.55)	2.00 ± 0.47	
Median	63		
ISS stage			
I	9 (24.32)	1.196 ± 0.18	0.0417∗
II	8 (21.62)	3.155 ± 0.80	
III	20 (54.06)	4.922 ± 1.42	
R-ISS stage			
I	9 (24.32)	1.2 ± 0.26	0.2056
II	20 (54.06)	3.02 ± 0.69	
III	8 (21.62)	5.27 ± 2.64	
M protein type			
IgG type	18 (48.66)	2.9 ± 0.78	
IgA type	6 (16.21)	1.12 ± 0.27	
IgM type	0		
Light chain type	7 (18.92)	2.88 ± 0.94	
Nonsecretary type	6 (16.21)	9.2 ± 3.79	

**Table 2:** Primer sequence of genes and siRNA.

Name	Sense (5′ to 3′)	Antisense (5′ to 3′)	Annealing temperature (°C)
lncRNA MSTRG.29039.1	GACGGAAACAGCCCTGCCTTC	CCCTCTCCCTCTCCTGTTGTGG	55
mRNA OSMR	ATTCCATTCCAGCACCAGCCAAC	GAAGCAGGAGAAGCACCCACAC	62
GAPDH	TGATGACATCAAGAAGGTGGTGAAG	TCCTTGGAGGCCATGTGGGCCAT	—
hsa-miR-12119	CGTTCTGAGGGGACGGTAGAT	AGTGCAGGGTCCGAGGTATT	55
hsa-miR-504-5p	GCGAGACCCTGGTCTGCAC	AGTGCAGGGTCCGAGGTATT	55
hsa-miR-658	GGCGGAGGGAAGTAGGTCC	AGTGCAGGGTCCGAGGTATT	55
RNU6A	CTCGCTTCGGCAGCACA	AACGCTTCACGAATTTGCGT	—
si-MSTRG	GGAUCGGAAAUGAGAAAUUTT	AAUUUCUCAUUUCCGAUCCTT	—
si-RNA	GUGCCUUUCAUUAGGAAUATT	UAUUCCUAAUGAAAGGCACTT	—
miRNA-inhibitor	CCCCAAAUCUACCGUCCCCUCAGAA		—
miRNA-NC	CAGUACUUUUGUGUAGUACAA		—
si-NC	UUCUCCGAACGUGUCACGUTT	ACGUGACACGUUCGGAGAATT	—

## Data Availability

The datasets used and/or analyzed during the current study are available from the corresponding author on reasonable request.

## Supplementary Materials

Supplementary Materials Supplementary Table 1: 63 differentially expressed lncRNAs in CD138+ cells of bone marrow in multiple myeloma. Supplementary Figure 1: (a) The relative expression level of hsa-miR-504-5p in MM patients and healthy controls by PCR. (b) Relative expression levels of hsa-miR-504-5p in AMO-1 and U266 cell lines before and after knockdowning si-MSTRG. (c) The relative expression level of hsa-miR-658 in MM patients and healthy controls by PCR. (d) Relative expression of hsa-miR-658 in AMO-1 and U266 cell lines before and after knockdowning si-MSTRG.
