# Supplementary material for: lncRNA MSTRG.29039.1 Promotes Proliferation by Sponging hsa-miR-12119 via JAK2/STAT3 Pathway in Multiple Myeloma
Source: Oxid Med Cell Longev. 2021 Aug 11;2021:9969449. doi: 10.1155/2021/9969449 (PMC8376436; doi:10.1155/2021/9969449)
Supplement: Supplementary Materials — Supplementary Table 1: 63 differentially expressed lncRNAs in CD138+ cells of bone marrow in multiple myeloma. Supplementary Figure 1: (a) The relative expression level of hsa-miR-504-5p in MM patients and healthy controls by PCR. (b) Relative expression levels of hsa-miR-504-5p in AMO-1 and U266 cell lines before and after knockdowning si-MSTRG. (c) The relative expression level of hsa-miR-658 in MM patients and healthy controls by PCR. (d) Relative expression of hsa-miR-658 in AMO-1 and U266 cell lines before and after knockdowning si-MSTRG. [file 9969449.f1.doc]

Supplementary table 1. 63 differentially expressed lncRNAs in CD138+ cells of bone marrow in multiple myeloma

| tracking_id | lncRNA_classify | Position | Strand |
| --- | --- | --- | --- |
| MSTRG.1916.2 | intergenic | chr1:149512484-149514241 | - |
| MSTRG.2641.2 | intergenic | chr1:192757399-192757673 | + |
| MSTRG.31117.1 | intergenic | chr9:69881034-69882441 | - |
| MSTRG.6369.3 | intergenic | chr12:4227247-4228241 | - |
| MSTRG.31039.3 | intergenic | chr9:66697106-66699457 | - |
| MSTRG.4878.1 | intergenic | chr11:9594456-9594833 | + |
| MSTRG.2874.1 | intergenic | chr1:209276403-209280920 | - |
| MSTRG.11201.1 | intergenic | chr15:100105222-100105480 | + |
| MSTRG.5876.1 | intergenic | chr11:87159254-87160265 | - |
| MSTRG.28963.8 | intergenic | chr7:115790598-115790751 | - |
| MSTRG.3570.1 | intergenic | chr10:22630911-22632371 | - |
| MSTRG.27271.1 | intergenic | chr6:109129242-109130174 | + |
| MSTRG.27693.5 | intergenic | chr6:156713709-156713843 | - |
| MSTRG.2630.1 | intergenic | chr1:189144430-189150386 | + |
| MSTRG.27985.1 | intergenic | chr7:12318951-12320457 | + |
| MSTRG.20669.1 | intergenic | chr22:20401449-20401687 | + |
| MSTRG.27552.1 | intergenic | chr6:140203579-140203824 | - |
| MSTRG.7819.1 | intergenic | chr12:116381229-116381729 | + |
| MSTRG.11479.1 | intergenic | chr16:3930528-3930760 | - |
| MSTRG.24095.1 | intergenic | chr4:121529663-121536190 | - |
| MSTRG.24960.1 | intergenic | chr5:70093788-70094114 | + |
| MSTRG.7820.1 | intergenic | chr12:116382641-116383361 | + |
| MSTRG.2300.1 | intergenic | chr1:160737094-160738188 | + |
| MSTRG.27396.1 | intergenic | chr6:125667557-125668180 | - |
| MSTRG.3807.1 | intergenic | chr10:52049587-52049861 | + |
| MSTRG.4162.1 | intergenic | chr10:88983375-88983894 | + |
| MSTRG.7285.1 | intergenic | chr12:67261441-67269879 | + |
| MSTRG.23651.1 | intergenic | chr4:68625608-68627326 | + |
| MSTRG.31852.1 | intergenic | chr9:134449033-134449680 | + |
| MSTRG.20184.1 | intergenic | chr21:15825244-15827452 | + |
| MSTRG.24983.1 | intergenic | chr5:70471482-70471939 | + |
| MSTRG.29039.1 | intergenic | chr7:126998503-127004645 | + |
| MSTRG.31076.1 | intergenic | chr9:68723581-68724090 | + |
| MSTRG.27830.1 | intergenic | chr6:170747024-170747623 | + |
| MSTRG.2992.1 | intergenic | chr1:222677723-222677943 | + |
| MSTRG.28019.1 | intergenic | chr7:17811294-17816274 | + |
| MSTRG.24980.1 | intergenic | chr5:70447429-70448001 | + |
| MSTRG.6750.2 | intergenic | chr12:27973721-27977057 | - |
| MSTRG.10770.1 | intergenic | chr15:70327558-70329565 | + |
| MSTRG.28352.1 | intergenic | chr7:50483116-50484601 | + |
| MSTRG.29317.1 | intergenic | chr7:149796553-149797730 | + |
| MSTRG.13656.1 | intergenic | chr17:38023516-38024237 | + |
| MSTRG.3574.1 | intergenic | chr10:22765985-22768762 | - |
| MSTRG.5886.1 | intergenic | chr11:88017826-88020849 | + |
| MSTRG.31071.1 | intergenic | chr9:68707813-68708503 | + |
| MSTRG.25534.1 | intergenic | chr5:135522002-135522211 | + |
| MSTRG.31075.1 | intergenic | chr9:68721408-68721716 | + |
| MSTRG.27410.1 | intergenic | chr6:126611930-126612999 | + |
| MSTRG.24975.1 | intergenic | chr5:70427776-70428126 | + |
| MSTRG.27752.1 | intergenic | chr6:160062890-160065533 | + |
| MSTRG.14893.1 | intergenic | chr18:23977223-23978627 | + |
| MSTRG.24977.1 | intergenic | chr5:70431272-70431836 | + |
| MSTRG.27231.1 | intergenic | chr6:106246275-106253976 | + |
| MSTRG.31214.1 | intergenic | chr9:82772007-82772815 | + |
| MSTRG.5011.1 | intergenic | chr11:26081921-26085013 | + |
| MSTRG.29958.1 | intergenic | chr8:64134698-64137165 | + |
| MSTRG.2644.1 | intergenic | chr1:192785814-192786969 | + |
| MSTRG.25423.1 | intergenic | chr5:127531684-127533518 | + |
| MSTRG.20807.1 | intergenic | chr22:23284436-23286082 | + |
| MSTRG.22142.1 | intergenic | chr3:81533416-81535755 | + |
| MSTRG.5810.1 | intergenic | chr11:80066117-80070596 | + |
| MSTRG.30059.1 | intergenic | chr8:82607541-82608932 | + |
| MSTRG.28347.1 | intergenic | chr7:50304725-50308532 | + |


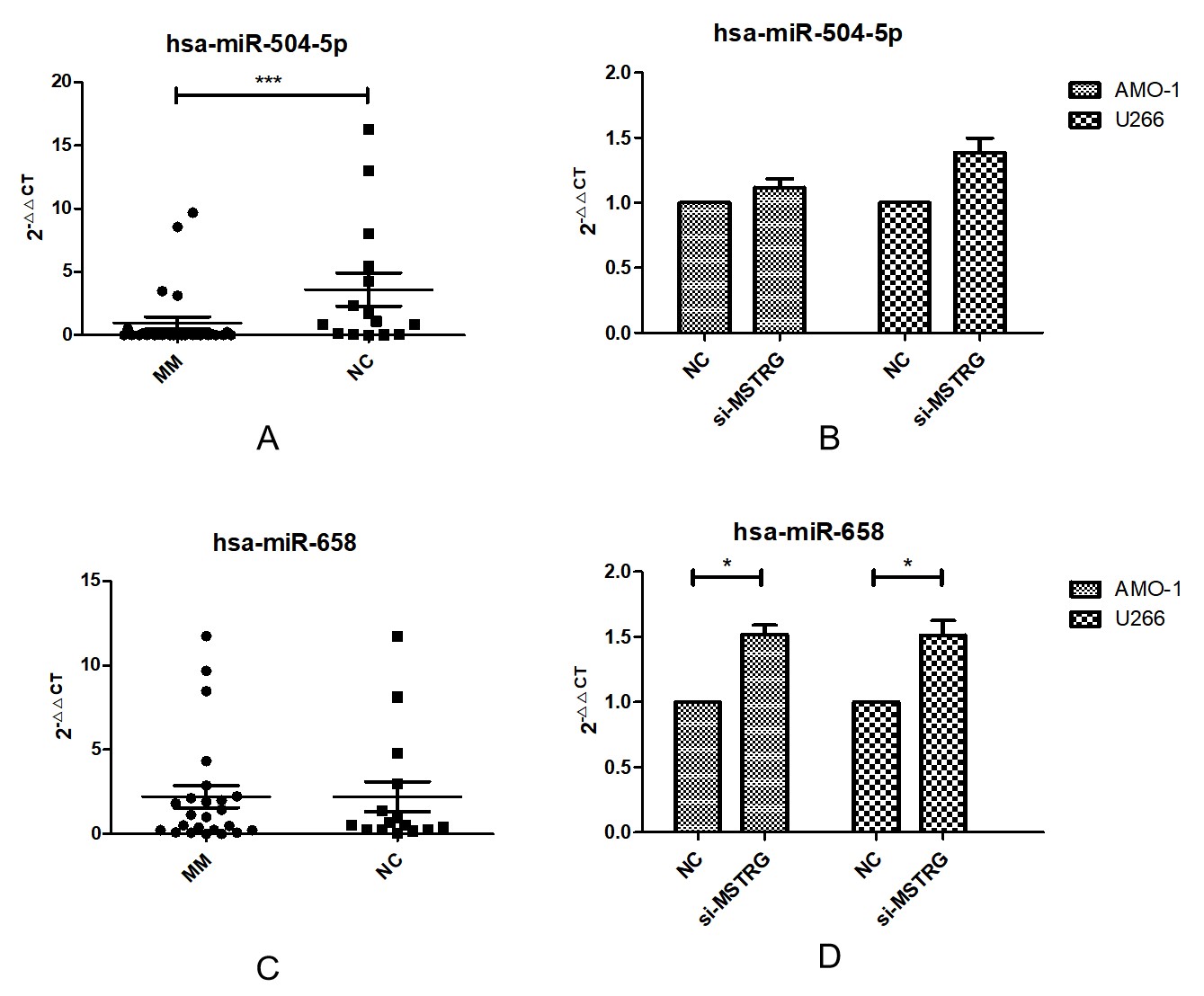


Supplementary figure 1.A. The relative expression level of hsa-miR-504-5p in MM patients and healthy controls by PCR; B. Relative expression level of hsa-miR-504-5p in AMO-1 and U266 cell lines before and after knockdowning si-MSTRG; C. The relative expression level of hsa-miR-658 in MM patients and healthy controls by PCR; D. Relative expression of hsa-miR-658 in AMO-1 and U266 cell lines before and after knockdowning si-MSTRG.
